# Intimidation and harassment in residency: a review of the literature and results of the 2012 Canadian Association of Interns and Residents National Survey

**Published:** 2014-12-17

**Authors:** Safiya Karim, Maryana Duchcherer

**Affiliations:** 1 2012–2013 CAIR Member Outreach Chair, Dept. of Internal Medicine, University of Saskatchewan, Saskatoon, Saskatchewan; 2 2012–2013 CAIR Member Outreach Committee, Dept. of Psychiatry, University of Alberta, Edmonton, Alberta

## Abstract

**Background:**

Intimidation and harassment (I&H) have been longstanding problems in residency training. These behaviours continue to be prevalent, as evidenced by the 2012 Canadian Association of Interns and Residents (CAIR) National Resident Survey. More than seven in ten (72.9%) residents reported behaviour from others that made them feel diminished during their residency. We conducted a literature review to identify other surveys to determine the prevalence, key themes, and solutions to I&H across residency programs.

**Method:**

PubMed and MEDLINE searches were performed using the key words “intimidation,” “harassment,” “inappropriate behaviour,” “abuse,” “mistreatment,” “discrimination,” and “residency.” The search was limited to English language articles published between 1996 and 2013, and to papers where ten or more residents were surveyed or interviewed.

**Results:**

A total of ten articles were reviewed. Our findings showed that I&H continue to be highly prevalent with 45–93% of residents reporting this behaviour on at least one occasion. Verbal abuse was the most predominant form; staff physicians and nurses tended to be the dominant source. Residents reported that I&H caused significant emotional impact; however, very few incidents of inappropriate behaviour were reported. Very few solutions to I&H were proposed.

**Conclusions:**

I&H in residency education continue to be common problems that are under-reported and under-discussed. The opportunity exists to improve efforts in this area. Definitions of what incorporates I&H should be revisited and various educational and structural initiatives should be implemented.

## Introduction

Intimidation and harassment (I&H) have been longstanding problems in residency training. Intimidation in medical education includes any behaviour, educational process, or tradition that induces fear or anxiety in the resident physician which generally has a detrimental effect on the learning environment.^1^ Furthermore, the Ontario Human Rights Code defines abuse and harassment as engaging in vexatious comments or conduct that is known or ought to be known to be unwelcome. Intimidation occurs when these words or actions disparage or humiliate the resident physician or cause the resident physician to undertake a course of action against his/her will, or refrain from undertaking an activity that, except for abuse or harassment, would be undertaken.^2^ Clearly, this is serious and potentially damaging.

The Canadian Association of Interns and Residents (CAIR) has been concerned with addressing intimidation and harassment issues in post-graduate medical education for a number of years. In 1996, CAIR published a position paper on intimidation and harassment.^1^ Since then, several efforts have been made to address I&H at the residency level. Many provincial resident organizations have published policies on this issue including a list of resources available to report I&H. At a national level, the Royal College of Physicians and Surgeons as well as the College of Family Physicians of Canada have included an I&H policy as part of their accreditation standards for residency programs.

Despite these efforts, I&H continue to be major issues for residents across Canada. In the 2012 CAIR National Resident Survey, more than seven in ten (72.9%) residents said they had experienced inappropriate behaviour from others that made them feel diminished during their residency. Half of all respondents (50.5%) said they had experienced this behaviour from either staff physicians or nursing staff. Over one quarter of respondents reported that they experienced yelling/shaming/condescension by colleagues (26.6%). Nearly four in ten (37.8%) cited their program director as a resource to help deal with inappropriate behaviour, and just over half (54.9%) said that the resources available to them were effective or somewhat effective.^3^

In order to better understand and address I&H, CAIR conducted a literature review to identify the prevalence, key themes and proposed solutions to I&H across residency programs.

## Methods

PubMed and MEDLINE searches were performed using the key words “intimidation,” “harassment,” “inappropriate behaviour,” “abuse,” “mistreatment,” “discrimination,” and “residency.” These search terms were used based on input from the 2012–2013 CAIR board of directors. The search was limited to English language articles published between 1996 and 2013. It was also limited to papers where ten or more residents were surveyed or interviewed.

Two individuals from the 2012–2013 CAIR Member Outreach Committee reviewed each article for inclusion. A total of ten articles met the above criteria and were agreed upon by both reviewers. The majority of the articles were based on surveys from residents in programs across the United States or Canada. Two articles were surveys of residents outside North America, namely from Japan and Nigeria. One article was based on a survey of graduates from family medicine residency programs.

Appendix 1 provides a summary of the articles reviewed in this document.

## Results

Eight major themes were discovered as part of the literature review, based on input from the two reviewers. These are listed below:

### 1) High prevalence of inappropriate behaviour

Resident intimidation, harassment and abuse continue to affect a significant number of residents. Eight of the ten studies reported that between 45–93% of residents had experienced some form of inappropriate behaviour during their residency training on at least one occasion.^3^,^4^,^6^,^8^–^12^ There did not seem to be significant variation in rates of intimidation and harassment between the North American and international residents.

### 2) Verbal abuse as the predominant form

In all the studies reviewed, verbal abuse was the most common form of intimidation and harassment experienced by residents. This was mostly in the form of inappropriate verbal comments and non-physical verbal threats.

Sexual harassment and gender discrimination were also noted as common forms of I&H in four studies.^4^,^6^,^8^,^11^ Sexual harassment was experienced by between 25–60% of residents, while one study from 1996 reported that 93% of residents surveyed had experienced some form of sexual harassment on at least one occasion.^11^ Female residents more commonly reported gender discrimination.^4^

### 3) Sources of abuse – physicians and nurses

All ten studies identified attending/staff physicians as a source of abuse. The next most common source was nursing staff, as identified in seven studies.^3^,^4^,^8^–^12^ Six studies also identified residents at higher levels as well as patients and their families as sources of abuse.^6^,^8^–^12^

### 4) Variation among rotations/specialties

Two studies looked at rates of I&H between various rotations or specialties. They both found that I&H were most common during a surgical rotation or in surgical specialties compared to non-surgical specialties.^6^,^8^ Of specific interest is the study by Musselman et al. (2005) that surveyed only surgical residents and faculty. This survey showed that many residents felt that “surgical culture” enabled them to accept behaviours that might otherwise be labeled as inappropriate. In surgical specialties I&H was rationalized: residents were more likely to classify behaviours as legitimate if they had a positive effect on their education.^5^

### 5) Cause of I&H – generation gaps and engrained culture

Two studies commented on the root cause of I&H in residency training. The Japanese study by Nagata-Kobayashi et al (2007) stated that negative traditions within the medical culture were the main cause of mistreatment.^6^ The 2006 American Medical Association survey concluded that “generation gaps in medicine create conflict that lends itself to behaviours of intimidation and harassment.”^7^

### 6) Negative Emotional Impact

Four studies discussed the emotional/workplace impact of I&H. These studies concluded that I&H generally had a negative impact on the residents’ work, that they experienced a decreased level of satisfaction with residency, and that anger and decreased eagerness to work were the most common emotional reactions to I&H.^6^,^8^–^10^

### 7) Awareness of reporting structures for I&H/reasons to avoid reporting

Five studies addressed the question of whether residents were aware of how to report I&H.^3^,^6^,^7^,^9^,^11^ These showed that between 50–75% of residents knew of the resources available to them to report inappropriate behaviours. However, only between 12–25% of incidents were actually reported. One study showed that of those residents that had reported I&H, 91% had experienced this behaviour on more than one occasion.^10^ In another survey, 50% of residents stated they did not feel comfortable reporting these behaviours to their residency program.^7^ Other reasons stated for not reporting the behaviours included that the individual did not think it was a problem, that they did not think that it was worthwhile, or that they did not believe that it would accomplish anything.^11^

### 8) Solutions to intimidation and harassment

Unfortunately, very few articles proposed specific solutions to dealing with I&H. Most studies proposed that residency programs should be charged with addressing these issues through prevention, education, identification and enforcement.^3^,^5^,^7^,^11^,^12^ The study by Cook et al. (1996) suggested several solutions via educational, behavioural and structural means. Incorporation of abuse and harassment topics in formal and informal curriculums as well as incorporating humanistic qualities in supervisor evaluations was suggested. Behavioural initiatives included labeling and addressing discriminatory and abusive events as well as issuing corporate policies in this regard. Structural solutions involved appointing a residency program ombudsperson, and establishing an institutional office to deal with problems of sexual harassment.^11^

## Discussion

The 2012 CAIR National Resident Survey and this literature review suggest that I&H in residency education continue to be common problems. Despite the implementation of several policies and resources to deal with inappropriate behaviour, residents often do not feel comfortable reporting these behaviours, believing that such actions would not lead to a favourable outcome or a change in behaviour. The problem, therefore, is not one of knowing that resources exist, but rather the lack of confidence in the effectiveness of these resources. This may be in part due to the fact that one of the major resources residents have identified is their program director. This may create an inherent conflict of interest, thereby deterring the resident from reporting inappropriate behaviours.

There are certain limitations to this paper. First, the review was limited to those papers that reported results of surveys of ten or more residents. By not including other types of literature, it is possible that certain themes within intimidation and harassment may have been omitted. In addition, potential solutions to the problem may not have been identified. Secondly, the results of this literature review are limited to the response rates of the surveys included. Two studies had a less than a 30% response rate.^3^,^7^ Finally, both reviewers were residents and members of the CAIR Member Outreach Committee. This may have introduced a bias into identifying themes based on their own residency experience.

I&H in residency education continue to be serious problems with high prevalence. The opportunity now exists for further work to be done in the area of I&H. Definitions of what incorporates inappropriate behaviour should be revisited and various educational and structural initiatives should be implemented. Further research needs to be conducted on effective solutions that are being used within and outside of the medical community. Of particular interest may be the establishment of a national or provincial residency ombudsperson(s) to eliminate the conflict of interest that may come from reporting inappropriate behaviours to a supervisor within the individual’s residency program.

## Figures and Tables

**Appendix 1:** Summary of articles reviewed

Author (Date)	Resident population	Methods	Results	Conclusions
**American Medical Association (2006)**	Residents in the US across all specialties	Cross sectional survey of 688 residents Response rate: 2.7%	Major themes: high prevalence of inappropriate behaviour, verbal abuse, sources of abuse, awareness of reporting structures, solutions to I+H 25% of respondents had non-physical harm threatened. The source of the threat was mainly from attending physicians. 68% of respondents stated that they knew how to report intimidation however 50% responded that they would not be comfortable reporting to their residency program.	Generation Gaps create conflict that lends itself to intimidation The consequences of intimidation are destructive to education and patient care Solutions to intimidation involve a multidirectional approach including: education, identification, and enforcement
**Canadian Association of Interns and Residents (2012)**	Residents across 13 Universities in Canada in all programs. Majority were 1^st^ to 3^rd^ year residents	Cross sectional survey of 2305 residents from 13 University programs across Canada in all specialties Response rate: 29.1%	Major themes: high prevalence of inappropriate behaviour, sources of abuse, awareness of reporting structures, solutions to I+H More than 7/10 residents reported some form of inappropriate behaviour that made them feel diminished. Female residents were more likely than male residents to experience inappropriate behaviour. 50% responded that the source of inappropriate behaviour was from a staff physician and 25% reported this behaviour from nursing staff. 38% of respondents stated that the program director was a resource to deal with inappropriate behaviour while 34% reported that there were no such resources available to them. Of those who reported that resources were available to them, 55% stated that these resources were effective.	Inappropriate behaviour is still experienced by the majority of residents on at least one occasion. Beyond their program director, residents remain unsure of resources available to them for dealing with inappropriate behaviour. There may be a need for more visible, confidential and dedicated resources to help residents in this area.
**Cohen, et al. (2008)**	Residents across Canada (except Quebec)	Cross-sectional survey of 1999 residents across 7 provinces. Response rate: 35%	Major themes: verbal abuse, sources of abuse, cause of I+H Residents reported intimidation and harassment most often from nursing staff (54%) followed by patients (45%) and staff physicians (39%). Most I&H was experienced in the form of verbal comments (66%). The perceived basis for the I&H was training status (30%) and gender (18%).	Although many residents have a positive outlook on their wellbeing, residents experience significant stressors and are at risk of emotional and mental health problems. Further research, advocacy and resource application is necessary.
**Cook, et al. (1996)**	Anaesthesia, internal medicine, family medicine, obstetrics and gynaecology, paediatrics, psychiatry and surgery residents	Cross sectional survey of 186 residents from 7 residency programs across 4 teaching hospitals associated with McMaster University Response rate: 82%	Major themes: high prevalence of inappropriate behaviour, sources of abuse, cause of I+H, awareness of reporting structures, solutions to I+H 93% of residents reported psychological abuse predominantly by supervising physicians, female nurses, patients and their families. 20% reported physical assault. The most common perpetrators were male patients and family members. 75% reported experiencing discrimination on the basis of gender. This was reported more by female residents. 93% reported at least one episode of sexual harassment. Of the residents who reported sexual harassment, 48% stated that they told someone about the event. Another resident, friend/partner or family member was most commonly cited as the confidante. Only 23% told a supervising physician. The reason most stated for not reporting sexual harassment was that the individual did not think the behaviour was a problem (46%). 25% thought that reporting the behaviour would not accomplish anything.	Psychological abuse, discrimination based on gender and sexual harassment are commonly experienced during residency training.
**Crutcher et al. (2011)**	Family medicine graduates from Alberta (University of Calgary and University of Alberta)	Retrospective questionnaire survey of 242 family medicine graduates between 2001–2005 Frequency, type, source and perceived source of intimidation, harassment and discrimination (IHD) Also asked if they were aware of process to address issues of IHD. Response rate: 64.2%	Major themes: high prevalence of inappropriate behaviour, verbal abuse, sources of abuse, cause of I+H, awareness of reporting structures, solutions to I+H 44.7% of graduates experiences IHD while a resident – 34% only once, 62% more than once. Inappropriate verbal comments most common form of IHD (94%), followed by work as punishment (28%). Main sources of IHD were from specialty physicians (77%), hospital nurses (54%), specialty residents (45%) and patients (35%). Primary basis of IHD was perceived to be gender (27%), ethnicity (16) and culture (10%). 54% of respondents knew about the process to address IHD during residency.	Perceptions of IHD are prevalent amongst family medicine graduates from Alberta. Residency programs should recognize and address any IHD concerns while actively promoting prevention.
**Daugherty, Baldwin, Rowley (1998)**	Random sample of all 2nd year residents listed in the American Medical Association medical research and information database	Cross-sectional survey of 1277 2nd year residents across the United States Response rate: 72%	Major themes: high prevalence of inappropriate behaviour, verbal abuse, sources of abuse, variation amongst rotations/specialties, negative emotional impact 93% of respondents noted at least 1 episode of perceived mistreatment during their internship year – most commonly in the form of humiliation or belittlement. 63% reported that mistreatment took place on 3 or more occasions. Attending faculty, residents at higher levels, patients and nurses were the most sited source of mistreatment. Sexual harassment or discrimination was reported to occur on at least 1 occasion by 30% of residents. Female residents experienced this behaviour more commonly than men. The main forms of sexual harassment or discrimination came in the form of sexual slurs or comments, followed by favouritism and sexual advances. The highest prevalence was in the surgical specialties (80%). Being humiliated or belittled had a high negative correlation with overall satisfaction with the first-year residency experience.	Residents report significant mistreatment during first year residency. Mistreatment was highly negatively correlated to overall satisfaction with first-year residency.
**Musselman et al. (2005)**	Surgical residents and faculty in 2 university departments of surgery	Group and individual interviews conducted across 2 university departments of surgery (22 faculty + 14 residents) Open ended questions regarding the definition of intimidation and harassment, followed by a discussion of 3 video scenarios that were based on the literature	Major themes: high prevalence of inappropriate behaviour, cause of I+H Participants were reluctant to use the terms intimidation and harassment. Participants felt that “surgical culture” allowed them to accept behaviours in the OR that in other circumstances they would label as I&H. There was a rationalization of “good intimidation” and that I&H was an effective learning tool suggested and supported by residents. If I&H could be linked to an acceptable purpose, it would more likely not be classified as I&H. Participants were more likely to classify behaviours as legitimate if they had a positive effect on education. Participants viewed I&H as both dysfunctional and functional.	Current definitions of intimidation and harassment are ambiguous. We must understand the functionality and dysfunctionality of I&H in surgical education, and what social circumstances cultivate these behaviours. We must devise educational alternatives that allow surgical teachers to achieve their core objectives while promoting a positive learning environment.
**Nagata-Kobayashi et al. (2007)**	Residents in Japan	Cross-sectional survey of 619 residents across 37 centers in Japan Prevalence of mistreatment in 6 categories was evaluated: verbal abuse, physical abuse, academic abuse, sexual harassment, gender discrimination and alcohol-associated harassment Response rate: 57.4%	Major themes: high prevalence of I+H, sources of abuse, variation amongst rotations/specialties, cause of I+H, negative emotional impact, awareness of reporting structures 85% of respondents reported mistreatment. Verbal abuse was most common (72%). Amongst women, sexual harassment was reported in 58% of respondents. Abuse was most likely to occur during the surgical rotation. Abusers were most often doctors, (35%) followed by patients (22%). Only 12% of respondents reported their experiences of abuse to superiors. The most common reason for not reporting was that is what thought not to be worthwhile, or would not accomplish anything. Anger and decreased eagerness to work were the most common emotional reactions to mistreatment (41% and 34% respectively).	Abuse and harassment during residency is a universal phenomenon. Negative traditions within the medical culture are the main cause of mistreatment. Strong preventative measures are needed.
**Ogunsemi, Alebiosu, Shorunmu (2010)**	Residents of a Nigerian teaching hospital	Single center survey of 58 residents Response rate: 80.6%	Major themes: high prevalence of inappropriate behaviour, verbal abuse, sources of abuse, negative emotional impact. 78% of residents reported experiencing intimidation and harassment through the course of the residency training. The source of I&H was from administration staff (58%), chief executive of the hospital (41%), patients families (40%) and from the nursing staff (33%). I&H were mainly in the form of inappropriate verbal comments (67%). Of those residents who had reported I&H, 91% had experienced I&H more than once.	Intimidation and harassment occurs among many residents. A number of residents are prone to emotional and mental health concerns during their training.
**Vanlneveld et al. (1996)**	Internal medicine residents in Canada	Cross-sectional survey of 543 residents across 13 programs Frequency of experienced and witnessed different types of abuse based on a 7-pt Likert scale How often they experienced psychological abuse, gender discrimination and sexual harassment How often they witnessed racial discrimination and homophobic remarks. Response rate: 84%	Major themes: high prevalence of inappropriate behaviour, sources of abuse Psychological abuse – Approximately equal frequency was attributed to attending physicians and nurses/other healthcare professionals (68–79%). Gender discrimination – Mostly experienced by females from their patients (47%), nurses/health care workers (36%) or by attending physicians (25%). Sexual harassment – Predominantly experienced by females from attending physicians, peers and patients. Equal rates of sexual harassment between males and females from nurses/healthcare workers. Physical assault – Almost exclusively from patients. Racial discrimination was commonly witnessed from patients (67%), peers (50%) and attending physicians (49%). Homophobic remarks were witnessed from all groups (53–61%).	Residency programs should start addressing prevention and management of bias, discrimination abuse by attending physicians and peers. Recommended curricular time to learn how to deal with abusive patients. These types of teachings should be incorporated into programs that already address resident stress

## References

[1] (1996). CAIR position paper on intimidation and harassment.

[2] Modified from the Ontario Human Rights Code.

[3] CAIR 2012 National Resident Survey.

[4] Vanlneveld C, Cook D, Kane S, King D (1996). Discrimination and abuse during internal medicine residency. J Gen Int Med.

[5] Musselman L, McRae H, Reznick R, Lingard L (2005). ‘You learn better under the gun’: intimidation and harassment in surgical education. Med Educ.

[6] Nagata-Kobayashi S, Maeno T, Yoshizu M, Shimbo T (2009). Universal problems during residency: abuse and harassment. Med Educ.

[7] 2006 American Medical Association Member Connect Survey AMA survey on issues of importance to resident physicians and fellows.

[8] Daugherty S, Baldwin D, Rowley B (1998). Learning, satisfaction and mistreatment during medical internship. JAMA.

[9] Cohen J (2008). The happy docs study: a Canadian Association of Interns and Residents well-being survey examining resident physician health and satisfaction within and outside residency training in Canada. BMC Res Notes.

[10] Ogunsemi O, Alebiosu O, Shorunmu O (2010). A survey of perceived stress, intimidation, harassment, and wellbeing of resident doctors in a Nigerian teaching hospital. Niger J Clin Pract.

[11] Cook D, Liutkus J, Risdon C, Griffith L, Guyatt G, Walter S (1996). Residents’ experiences of abuse, discrimination and sexual harassment during residency training. CMAJ.

[12] Crutcher R, Szafran O, Woloshuk W, Chatur F, Hansen C (2011). Family medicine graduates’ perceptions of intimidation, harassment and discrimination during residency training. BMC Med Educ.

